# Methane Production Rate during Anoxic Litter Decomposition Depends on Si Mass Fractions, Nutrient Stoichiometry, and Carbon Quality

**DOI:** 10.3390/plants10040618

**Published:** 2021-03-24

**Authors:** Annkathrin Hömberg, Klaus-Holger Knorr, Jörg Schaller

**Affiliations:** 1 Ecohydrology & Biogeochemistry Group, University of Münster, Heisenbergstraße 2, 48149 Münster, Germany; kh.knorr@uni-muenster.de; 2 Leibniz Center for Agricultural Landscape Research (ZALF), 15374 Müncheberg, Germany; Joerg.Schaller@zalf.de

**Keywords:** Silicon, *Eriophorum vaginatum*, peatland, organic matter degradation, carbon quality, biogenic silica

## Abstract

While Si influences nutrient stoichiometry and decomposition of graminoid litter, it is still unclear how Si influences anoxic litter decomposition and CH_4_ formation in graminoid dominated fen peatlands. First, *Eriophorum vaginatum* plants were grown under different Si and P availabilities, then shoots and roots were characterized regarding their proportions of C, Si, N and P and regarding C quality. Subsequently the *Eriophorum* shoots were subjected to anoxic decomposition. We hypothesized; that (I) litter grown under high Si availability would show a higher Si but lower nutrient mass fractions and a lower share of recalcitrant carbon moieties; (II) high-Si litter would show higher CH_4_ and CO_2_ production rates during anoxic decomposition; (III) methanogenesis would occur earlier in less recalcitrant high-Si litter, compared to low-Si litter. We found a higher Si mass fraction that coincides with a general decrease in C and N mass fractions and decreased share of recalcitrant organic moieties. For high-Si litter, the CH_4_ production rate was higher, but there was no long-term influence on the CO_2_ production rate. More labile high-Si litter and a differential response in nutrient stoichiometry led to faster onset of methanogenesis. This may have important implications for our understanding of anaerobic carbon turnover in graminoid-rich fens.

## 1. Introduction

During the postglacial period, northern peatlands began acting as great carbon (C) sinks, now comprising a C pool of >500 Gt of C [1], while covering only about 3% of the earth’s land surface [2]. This C accumulation occurs because once grown organic matter reaches the permanently waterlogged, anoxic parts of the profile, the catotelm, decomposition proceeds much more slowly than under oxic conditions of the acrotelm [3]. Despite the function as C sink, peatlands are also a source of greenhouse gasses such as carbon dioxide (CO_2_) and methane (CH_4_) [4]. In groundwater-fed peatlands, i.e., fens, the predominant vegetation is dwarf shrubs and graminoids, whereby graminoid roots primarily form the peat [5]. The aboveground biomass, which decomposes faster, is predominantly involved in respiration and methanogenesis and hence CH_4_ and CO_2_ production [5].

Decomposition of peat, which governs the release of dissolved organic carbon (DOC) and mineralization to CO_2_ and CH_4_, depends on the prevailing biogeochemical conditions [4], most importantly, the microbial activity, nutrient availability, electron acceptor availability, and the quality of the organic matter [4]. The nutrient availability in the peat influences decomposability [6] and the nutrient content in standing biomass [7,8], which after senescence undergoes decomposition and recycling. For a specific plant species, higher nutrient content can lead to lower amounts of microbially immobilized nitrogen (N) and phosphorus (P) and a faster release of N and P from decomposing litter [6]. This effect was amplified by higher litter production on plots with a higher N and P supply. However, across different plant species, a higher nutrient supply did not necessarily lead to higher decomposition rates [6].

One nutrient that has often been characterized as “beneficial” but “not essential”, is silicon (Si). In wetland graminoids, Si may make up >5% of the dry mass [9], but Si is still not considered “essential”, because a lack of Si does not prevent plants from completing their life cycle nor is Si an essential plant metabolite [9,10]. Plants deprived of Si are often weaker, though they are hampered in their development and are prone to abiotic and biotic stresses [11]. Moreover, studies have repeatedly shown that Si accumulation in rice also influences the carbon quality in the litter [12,13,14]. Schaller et al. [11] found that litter with higher Si mass fractions had a lower share of cellulose, lignin, phenol, fat, wax, lipids and free organic acids, most of which are considered more recalcitrant compounds in plant litter [5]. As Si clearly influences living biomass and causes qualitative changes in the carbon compounds, the question arises as to whether the Si mass fraction, and thereby, its influence on C quality and nutrient stoichiometry, also affect the decomposition of litter from plants commonly found in fens.

The role of Si in plant litter decomposition was investigated by Schaller and Struyf [12] for *Phragmites australis* (Cav.) Trin. ex Steud rich and poor in Si. They based their experiment on the observation made by McClaugherty and Berg [8], who found that litter decay is influenced by the N and P content, the content of hardly degradable compounds like lignin and cellulose, and the phenol content. They found that a higher nutrient content and a lower content of more recalcitrant compounds and phenols increased decomposition rates [12]. Further, since Si influences the nutrient stoichiometry and, e.g., the share of phenols, as shown above, the authors proposed Si may directly or indirectly influence the decomposition of grasses. Contrary to the authors’ expectations, Schaller and Struyf [12] found the highest rates of microbial decomposition under high Si availability. According to their interpretation, this was because high-Si conditions resulted in lower shares of cellulose and phenolics, and because silicic acid may have promoted fungal growth [13]. A higher Si mass fraction may go along with decreased nutrient mass fractions [12], but since the carbon quality is also changed under high Si uptake [11], the litter is probably more labile than litter with a lower Si mass fraction. Therefore, these effects may outweigh or interfere with each other and this must be further examined.

Under anoxic conditions and in absence of vegetation, an increase of CO_2_ and CH_4_ concentrations in peat pore waters were found, accompanied by a narrower CO_2_:CH_4_ ratio [14]. Mobilized P was suggested to have stimulated microbial degradation of DOC, leading to increased CH_4_ emissions. Si may have also exerted a direct influence on decomposition, though in this earlier study no mechanism was suggested. Nonetheless, the decreased mass fractions of P in graminoid litter due to the increased Si content promoted decomposition, as confirmed in other research [12].

Regardless of these uncertainties, most studies, with few exceptions, examined the effects in oxic environments. However, in wetlands and fens, decomposition in the anoxic peat is important, as this may control the formation of CH_4_ as an important greenhouse gas from anoxic wetland soils. However, we lack studies that specifically address anaerobic decomposition of litter differing in Si mass fractions. Therefore, we conducted a comprehensive study on; (1) the influence of increased Si availabilities in peat on the nutrient mass fractions and carbon quality in above- and belowground litter of *Eriophorum vaginatum* L.; and (2) on the differences in anaerobic decomposition of *Eriophorum vaginatum* aboveground litter differing in mass fractions of Si. In the first part of the study, we conducted a plant growth experiment with *Eriophorum vaginatum* under three levels of Si availability in the substrate. To decouple the effect of Si and the effect of P, we also integrated treatments with two different levels of phosphate fertilization. For the second part of the study, the litter of *Eriophorum vaginatum* that was grown under these conditions was subjected to decomposition in an incubation with six types of *Eriophorum vaginatum* aboveground litter.

For the Si and P fertilization experiment we hypothesized that; (I) litter grown under high Si availability in the substrate would have lower nutrient mass fractions and a lower share of recalcitrant compounds. For the decomposition experiment, we expected that, (II) litter with a high Si mass fraction would show higher CH_4_ and CO_2_ production rates upon anoxic incubation due to a lower share of recalcitrant organic matter. Moreover, we hypothesized that; (III) the onset of methanogenesis would be earlier for the decomposition of litter high in Si, compared to litter low in Si due to fast depletion of electron acceptors under high Si availability.

## 2. Results

### 2.1. Part I—Characterization of the Plant Material

#### Stoichiometry

**Figure A1:**
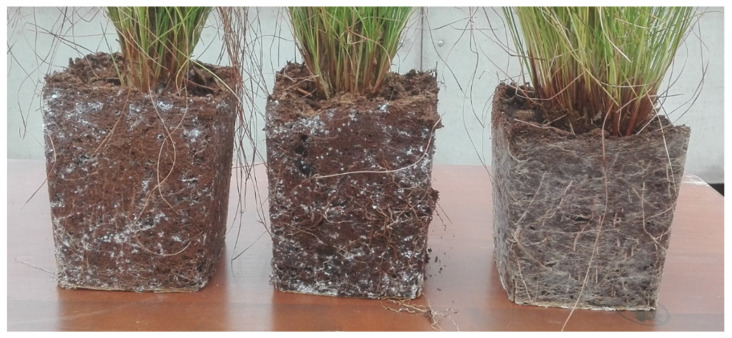
Soil of the *Eriophorum* plants prior to harvest. Left side: substrate contains 0% SiO_2_, Middle: substrate contains 1% SiO_2_, Right side, substrate contains 10% Si. White points in the left and middle sample are root aphids (*Rhizoecus* ssp.). The Si 10% treatment showed no infestation.

*Carex* individuals produced very little biomass, especially aboveground. This did not visibly change with treatment. *Eriophorum* individuals were more vigorous in growth. Individuals that received more P with fertilization appeared more vital. By visual inspection, those plants that grew in the Si-rich substrate tended to develop more aboveground biomass, but as this was not the focus of our study, the exact biomass was not determined. Visual examination of the substrate during harvest revealed an infestation with root aphids (*Rhizoecus* ssp.) in the no-Si and the low-Si treatment, but not in the Si 10% treatment. The root biomass was substantially higher in the Si 10% treatment compared to the other treatments (Figure A1).

1. *Eriophorum* shoots

For detailed accounts of statistical data in brackets, we code the treatments as follows:
**Low-P Si 0%****Low-P Si 1%****Low-P Si10%****High-P Si 0%****High-P Si 1%****High-P Si10%**‘1’‘2’‘3’‘I’‘II’‘III’

**Figure 1:**
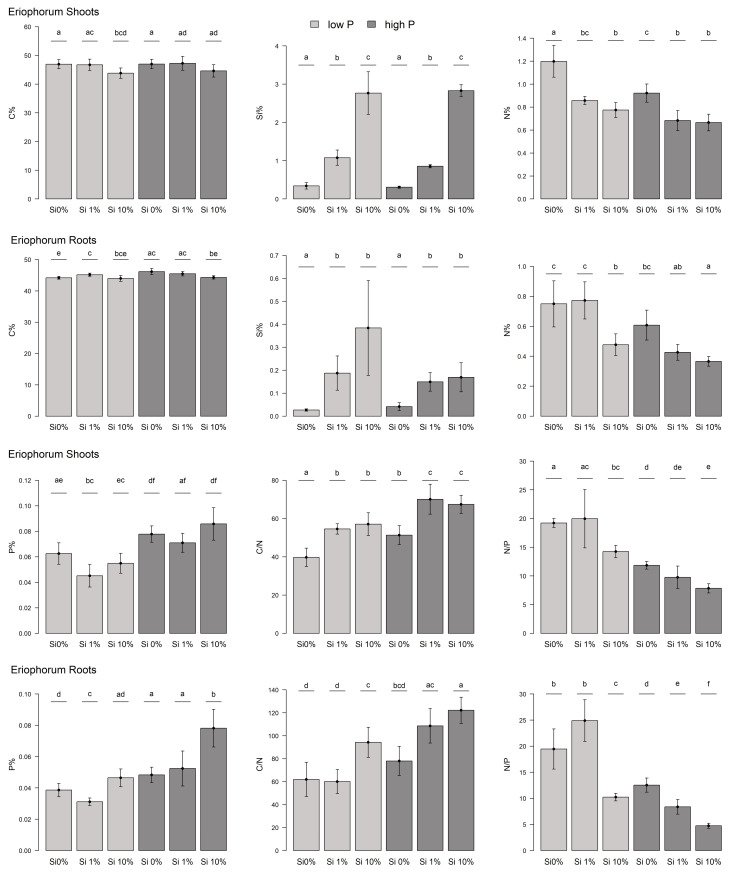
Element mass fractions and stoichiometry of *Eriophorum* aboveground and belowground biomass. Mass fractions of C, Si, N as well as P and the C/N and N/P ratios are displayed. Low-P fertilized plants: light gray, high-P fertilized plants: dark gray. Different letters give significant differences between the treatments (α = 0.05).

**Figure 2:**
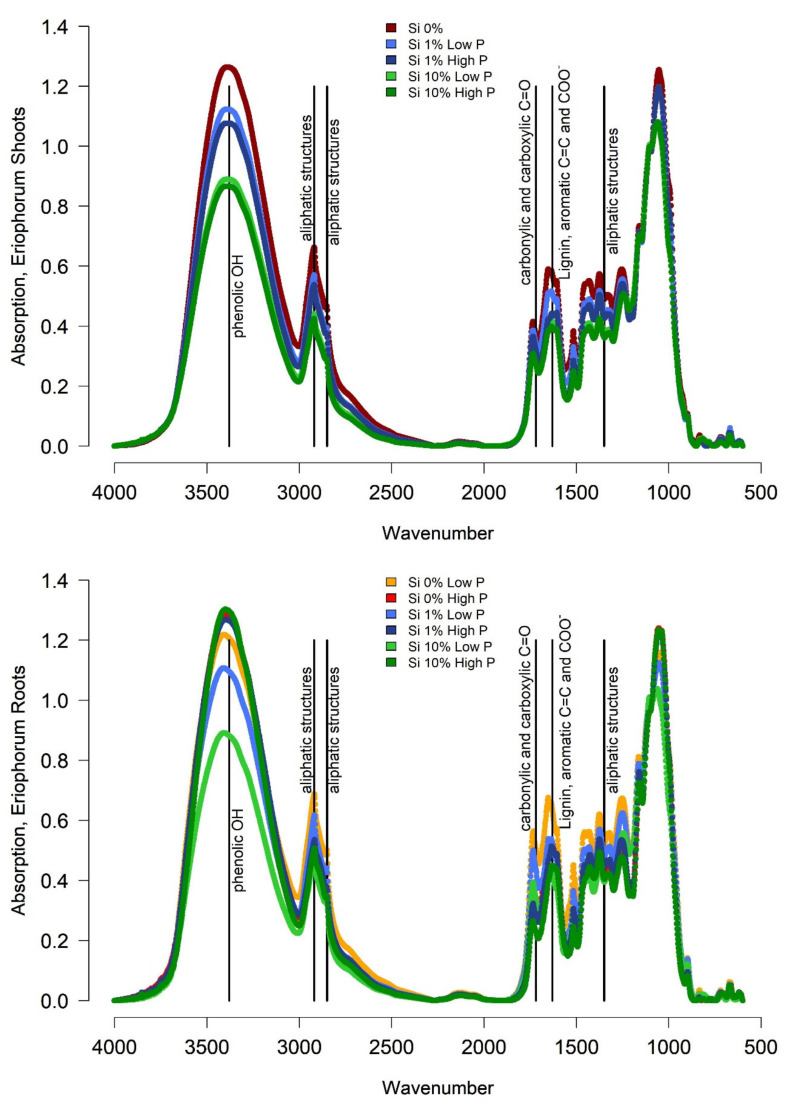
FTIR Spectra of *Eriophorum* shoots and roots before the incubation. For *Eriophorum* shoots only a mixed sample of the Si 0% low and Si 0% high P treatment is available (divergent color code: Si 0%: dark red).

We found lower C mass fractions in the shoots of *Eriophorum* for those treatments that had increased amounts of Si in the substrate (Figure 1). The difference in the C mass fraction between the “low-P Si 0%” and “low-P Si 10%” treatments was significant (*t*-test, *p*-value = 0.03). There was no difference in the C mass fractions between the low-P and high-P treatment groups. The mass fractions of C varied between 41% and 49% of the dry mass. Compared to the *Carex* shoots, the C mass fraction of *Eriophorum* was significantly higher (Figure 2).

**Figure A2:**
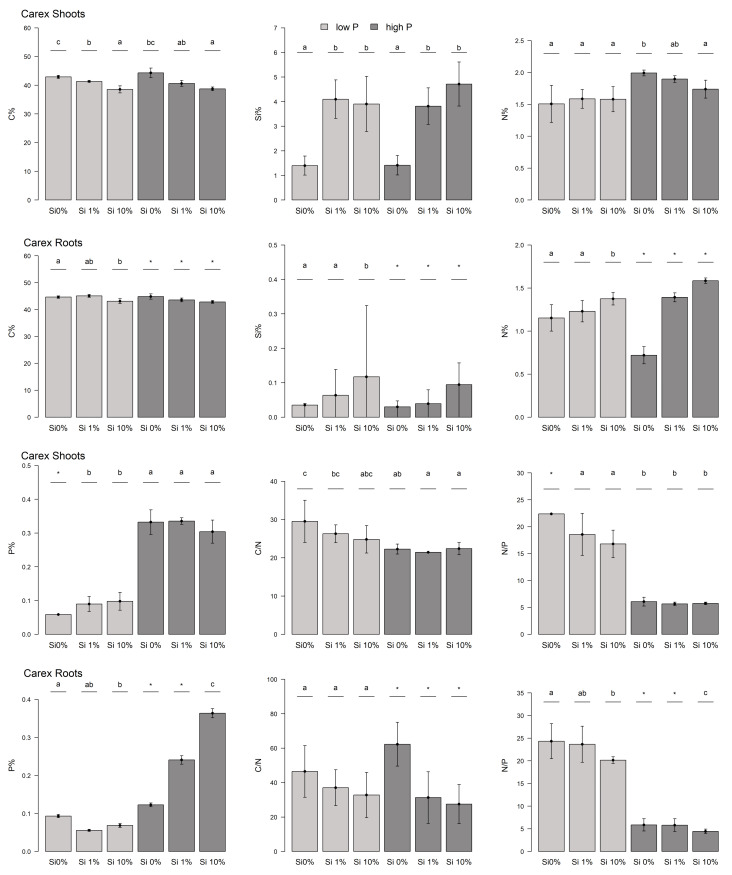
*Carex* aboveground and belowground biomass. Contents of C, Si, N as well as P and the C/N and N/P ratios are displayed. Low P fertilized plants: light gray, high P fertilized plants: dark gray. Different letters give significant differences between the treatments (α = 0.05), those treatments that are marked with an asterisk (*) have too few data for statistical evaluation.

The mass fraction of Si increased with increasing Si addition to the substrate (Figure 1). The differences between the different Si treatments were significant (All: *t*-test: 1 and 2: *p* = 1.141 × 10^−4^/1 and 3: *p* = 8.833 × 10^−4^/2 and 3: *p* = 4.621 × 10^−4^/I and II: *p* = 1.868 × 10^−8^/I & III: *p* = 2.813 × 10^−6^ /II & III: *p* = 4.765 × 10^−6^). Yet, there was no difference in the Si mass fraction of the shoots, between the low-P and high-P treatment groups. *Carex* shoots had a tendentially higher Si content compared to *Eriophorum* shoots, especially in the low-P treatment (not significant; Figure A2).

The mass fraction of N in the Si 0% shoots was significantly higher compared with those treatments with Si added to the substrate (All: *t*-test: 1&2: *p* = 0.006/1&3: *p* = 5.419 × 10^−4^/I&II: *p* = 3.669 × 10^−3^/I&III: *p* = 1.399 × 10^−3^) (Figure 1). The difference between Si 1% and Si 10% was not significant, but the decreasing trend was apparent. Moreover, the high-P treatment group here had a significantly lower N mass fraction, compared to the low-P treatment group (*t*-test, *p*-value = 0.004). The N mass fraction was higher for *Carex* compared to *Eriophorum* in most treatments (Figure A2).

There was more P in the shoots that received higher P fertilization (*t*-test, *p*-value = 0.009) compared to low-P (Figure 1). We found that there was no clear trend in the P mass fraction regarding the different Si additions, except for a significantly (“low-P Si 1%”) or insignificantly (“high-P Si 1%”) lower value compared to the Si 0% and the Si 10% treatments.

The C/N ratio was significantly higher for those treatments that had higher Si availability in the substrate compared to the Si 0% treatment (All: *t*-test: 1&2: *p* = 6.52 × 10^−4^/1&3: *p* = 1.913 × 10^−3^/I&II: *p* = 3.684 × 10^−3^/I&III: *p* = 1.55 × 10^−3^) (Figure 1). This was due to the decreasing C mass fraction with increasing Si availability and the differently changing N mass fraction. The C/N ratio of *Carex* shoots was significantly lower than that of *Eriophorum* (Figure A2).

The N/P ratio had a decreasing trend with increasing Si availability, albeit mostly not significant (Figure 1). The Si 0% and the Si 10% treatments differed in N/P in both P treatment groups, respectively. The N/P ratio was significantly lower for the high-P treatment group, compared to the low-P treatment group (*t*-test, *p*-value = 0.003).

*Eriophorum* shoots of the Si 0% treatment showed the highest absorption signal compared to the other treatments around wavenumber 3380, indicating phenolic structures and around wavenumbers 1630, 2850 and 2920, indicating aliphatic structures (Table 1). Moreover, a higher share of lignin (wavenumber 1630) was found. The treatments with Si 1% showed lower absorption than the Si 0% treatment and the Si 10% treatment showed even lower absorptions. The high P treatment showed a slightly lower signal respectively, especially around wavenumber 3380. The fourier transform infrared spectroscopy (FTIR) ratios (Table A3) of the *Eriophorum* shoot material showed comparable patters. All ratios showed decreasing values with the increasing Si mass fraction in the leaves in both, the low and high P treatment group.

2. *Eriophorum* roots

The roots of *Eriophorum* (Figure 1) showed similar patterns to its aboveground biomass. Generally, the mass fraction of C decreased slightly with increasing Si availability. This difference in C was significant comparing “high-P Si 0%” and “high-P Si 10%” (*t*-test, *p* = 9.673 × 10^−3^). In the low-P treatment group, the Si 1% treatment was an exception, with the C mass fraction significantly higher compared to the Si 0% treatment (*t*-test, *p* = 0.021). No general difference was found from the mass fraction of C in the *Eriophorum* shoots.

The Si mass fraction was much lower (0.02% to 0.64%) in the *Eriophorum* roots than in the shoots of *Eriophorum*, though (0.27% to 3.81%) (Figure 1). The Si 1% and Si 10% treatments had, as expected, significantly more Si than the Si 0% treatments (*welch*-test: 1&2: *p* = 0.012/*welch*-test: 1&3: *p* = 0.026/*t*-test: I&II: *p* = 1.186 × 10^−3^/*welch*-test: I&III: *p* = 0.014).

In relation to N, there was no significant difference between the “low-P Si 0%” and the “low-P Si 1%” treatment, whereas the “low-P Si 10%” treatment displayed a significantly lower mass fraction of N (1&3: *t*-test, *p* = 0.123/2&3: *t*-test, *p* = 3.295 × 10^−3^) (Figure 1). For the high-P treatment this holds true comparing the “high-P Si 0%” and the “high-P Si 10%” treatment (*t*-test, *p* = 1.706 × 10^−3^). Besides the “low-P Si 1%” treatment, all treatments had a significantly lower N mass fraction in the *Eriophorum* roots than in the shoots (Figure 1).

For the *Eriophorum* roots, the mass fraction of P was lower for the Si 0% and the Si 1% treatments of both P fertilization variants, compared to the shoots (Figure 1). The P mass fraction generally increased slightly with increasing P availability.

Due to the significant effect of Si availability on the N mass fraction in the Si 10% treatment (see above), the C/N ratio was significantly higher for the “low-P Si 10%” treatments compared to the ”low-P Si 0%” and the “low-P Si 1%” treatment (1&3: *t*-test, *p* = 0.012/2&3: *t*-test, *p* = 3.511 × 10^−3^) (Figure 1). The difference in the C/N ratio between the “high-P Si0%” and the “high-P Si10%” treatments was also significant (I&III: *t*-test, *p* = 8.474 × 10^−4^).

The N/P ratio displayed large differences between the treatments, which was due to the described patterns in N and P (Figure 1). The ratio decreased with increasing Si availability and was also lower for the high-P treatment group due to the low N mass fraction.

The roots of *Eriophorum* displayed slightly higher FTIR ratios than *Eriophorum* shoots (Table A3). The differences between Si treatments were much less clear (Figure 2). Instead, there was a difference in the absorption between the low-P and the high-P treatments, the latter always showed a higher absorption at wavenumbers, which was indicative of more refractory compounds. This difference was especially pronounced in the “Si 10%” treatment.

### 2.2. Decomposition of the Plant Material

#### 2.2.1. CO_2_ and CH_4_ Production Rates of Eriophorum Shoot Litter

**Figure 3:**
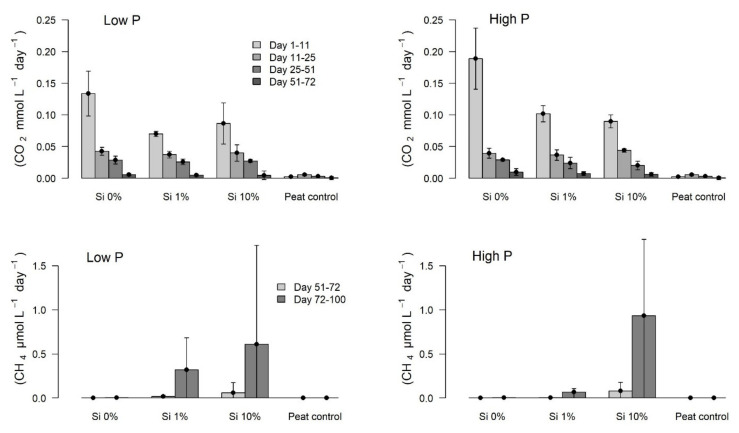
CO_2_ (top) and CH_4_ (bottom) production rates during the initial phase (day 1–11) and the long-term incubation period of *Eriophorum* shoots. Methanogenesis only started after 50 days, therefore the time before is not displayed in the graphs at the bottom.

Within the first 11 days, the CO_2_ production rate was significantly higher in the Si 0% treatments compared to the Si 1% treatment in the low-P treatment group and compared to the Si 1% and Si 10% in the high-P treatment group (all: *welch*-test: 1&2: *p* = 0.022/I&II: *p* = 0.020/I&III: *p* = 0.013). The CO_2_ production of the control (peat only) was significantly smaller than production rates of all other incubations with shoot biomass (Figure 3).

In the following phases of the long-term incubation, i.e., day 11–25, day 25–51 and day 51–72, there were no significant differences in the CO_2_ production rates between the treatments, except the control without litter, producing CO_2_ at a significantly lower rate. During the incubation, the mean CO_2_ production rate (except the control) decreased substantially. From day 72–100, the CO_2_ production stagnated at a very low level.

The CH_4_ production (Figure 3) only began after about 50 days of incubation. The variance of this time lag was large, as some bottles produced CH_4_ early (specially from the Si 10% group) and others late. Due to the number of replicates (5) this led to hardly any significant differences. Nevertheless, the tendency was apparent: A higher Si mass fraction in the shoots led to higher CH_4_ production rates in the incubation. This held true for both P treatment groups and both displayed CH_4_ production only in the later phase of the 100 days incubation during day 51–72 and day 72–100. At the end, CH_4_ production was on average 10-fold larger in the high-Si treatments than during day 51–72. The Si 0% treatment however, showed hardly any CH_4_ production throughout the 100-day incubation.

#### 2.2.2. Litter Quality and C and N Mass Fractions of Eriophorum Shoots after Incubation

**Figure 4:**
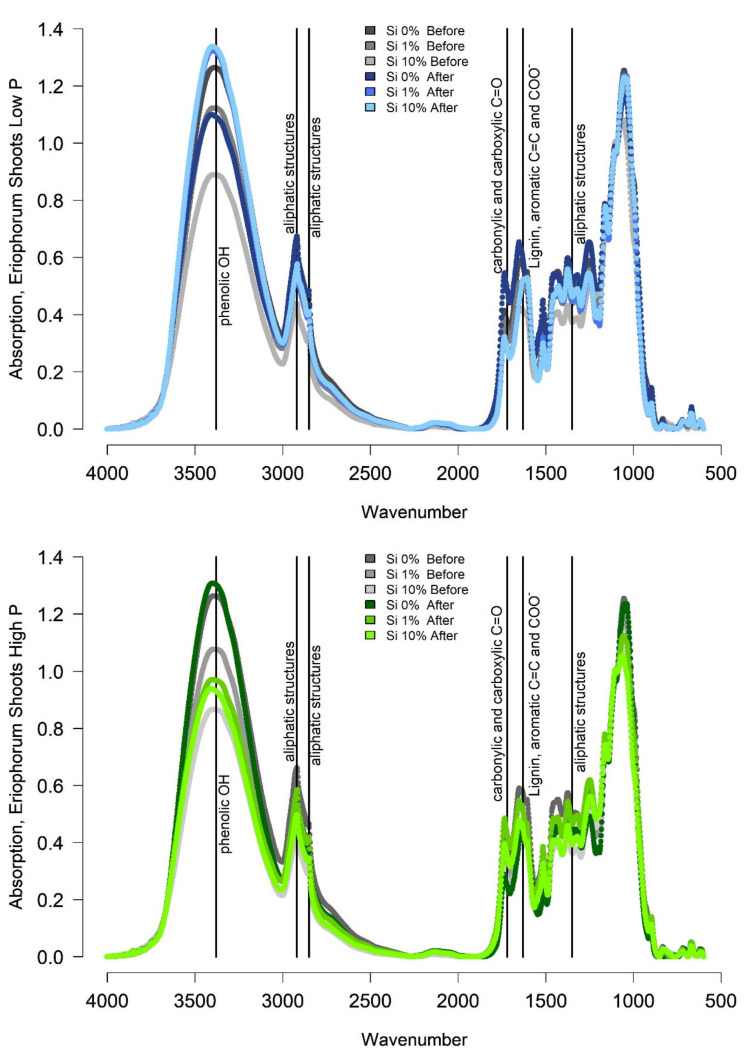
FTIR Spectra of *Eriophorum* shoots after the incubation in blue (low-P treatments) and green (high-P treatments). For the Si 0% both, the low and the high P spectrum, are the same, since no individual samples were available. Correspondent spectra from before the incubation are given in gray for orientation. The brightness of colors is according to the Si treatment.

The shoots of *Eriophorum* after incubation differed in their FTIR signal to the shoots before the incubation (Figure 4).

The absorption spectra, especially around wavenumber 3380, were higher for the “low-P Si 1%” and the “low-P Si 10%” treatment after the incubation compared to before due to a relative increase in the share of recalcitrant material (Figure 4). The “Si 0% low-P” spectrum rather showed a lower absorption around wavenumber 3380 after the incubation. For the high-P treatment group, the Si 0% and the Si 10% treatment showed higher absorption around wavenumber 3380, only the Si 1% spectrum showed lower absorption after the incubation compared to before the incubation. Meanwhile, most ratios increased or decreased in the same manner, displaying a higher share of recalcitrant material after the incubation for the “low-P Si 1%”, the “low-P Si 10%” and the “high-P Si 0%” and the “high-P Si 10%” treatment.

**Figure 5:**
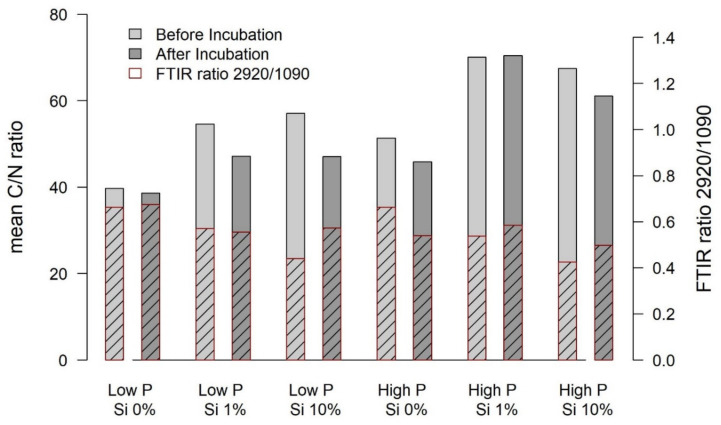
Mean values of the C/N ratios of biomass of *Eriophorum* shoots before and after the incubation (left *y*-axis). The hatched area denotes the FTIR ratio 2920/1090 (indicative for aliphatic structures; waxes and lipids) before and after the incubation—referring to the right axis.

The C mass fraction of *Eriophorum* shoots generally decreased over the incubation period. However, the “low-P Si 10%” treatment showed an increasing C mass fraction over the incubation period. The N mass fraction increased in the Si addition treatments of the low-P group and in the “high-P Si 0%” treatment and did not differ for the other treatments. The C/N ratio (Figure 5) generally decreased over the incubation time, as expected, but showing no significant difference for the “low-P Si 0%” and the “high-P Si 1%” treatment. 

#### 2.2.3. Nutrient Leaching into the Water Phase

**Figure 6:**
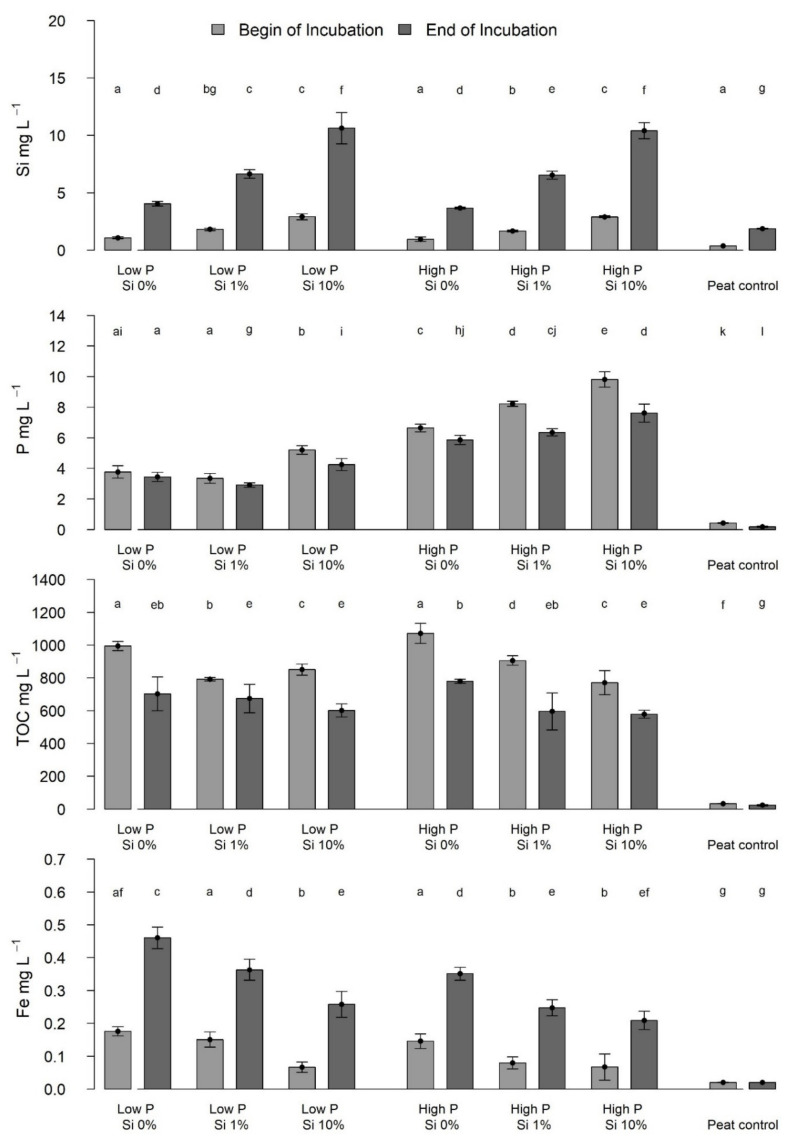
Concentrations of dissolved Si, P, TOC and Fe in mg L^−1^ in the water phase after 11 days (light grey bars) and after 100 days (dark gray bars) at the begin and end of *Eriophorum* shoot incubation. Lower case letters indicate significant differences (*p* < 0.05).

The concentrations of dissolved Si, P, TOC, and Fe in the water, at the beginning and at the end of the incubation with *Eriophorum* shoots, displayed significant differences (Figure 6) (for detailed statistics see Table A4). The concentrations of Fe and Si were significantly higher at the end of the incubation for all treatments.

In comparing the concentration differences of Si, TOC, P and Fe at the beginning and the end of the incubation, significantly higher (Figure 6, Table A4) differences were found in the Si concentrations of the “Si 10%” treatments than for the other treatments. The P treatment did not influence the Si concentration increase. Concentrations in Fe increased more strongly in the low-P compared to high P treatments.

The concentration of TOC and P decreased over time; TOC concentrations were significantly lower (Figure 6, Table A4) in each treatment group, and P concentrations were lower in each treatment except the “low-P Si 0%” treatment group. In the high-P treatments, the P concentration, and the decrease in concentration during the experiment in the water phase was significantly higher than in the low-P treatments (Figure 6, Table A4). The decrease in P concentration during the experiment was larger for the high-Si treatments.

## 3. Discussion

### 3.1. Characterization

Our study revealed some general differences between the investigated species concerning Si uptake and its effects on other nutrients stoichiometry. *Carex* shoots had a higher Si mass fraction (0.8–5.6% _dm_) than *Eriophorum* shoots (0.3–3.8% _dm_), and *Carex* shoots accumulated more Si especially in the 1% Si treatment, which reached an Si mass fraction in the shoots as high as in the 10% Si treatment. Both values were on the same order of magnitude as for rice straw investigated by Klotzbücher et al. [17] (3.7 ± 1.2% standard deviation) and coincide with Si contents reported for graminoids [18,19]. Compared to our field survey data on *Eriophorum* Si mass fractions, the 0% Si treatment had an Si mass fraction just slightly below these field data (0.5–0.6% Si in *Eriophorum* shoots from the field).

*Carex* had a generally higher N mass fraction compared to *Eriophorum* in the shoots and roots. Klotzbücher et al. [17] found the Si content to be severalfold higher than the N and P contents of the rice straw they investigated. Here, we only found this to be true for the shoots of both species that were amended with at least 1% Si in the substrate. The roots always contained less Si than N on a mass basis. *Eriophorum* showed decreasing mass fractions of N with increasing P availability and *Carex* displayed a higher N mass fraction under increasing P availability. *Carex* accumulated much more P compared to *Eriophorum* when high amounts of P were available. Therefore, the N/P and the C/N ratio differed between both species. *Carex* generally had a narrower C/N ratio compared to *Eriophorum*, and for *Carex* the N/P ratio showed stronger dependence on the P level.

Based on reported observations [11,17], we hypothesized; that (I) litter grown under high Si availability in the substrate would show lower N and P mass fractions and a lower share of phenols, aliphatic groups, carboxylic groups, and waxes. Concerning *Eriophorum* shoots, this hypothesis was verified for N, but not for P. The C mass fraction also decreased with increasing Si content of the shoots. Both findings align with the findings of some studies [19,20], but contrast with others in which N uptake correlated positively with Si uptake for *poaceae*.

However, Schaller et al. [20], found N did not correlate with Si in rice straw. Also, we found that the P mass fraction did not depend on Si content in *Eriophorum* shoots, coinciding with the findings of Klotzbücher et al. [17]. *Eriophorum* roots showed a generally similar pattern, where Si had a significant influence on the N mass fraction, especially in the high Si treatment. When the Si mass fraction was higher, the roots’ C/N ratio was consequently higher and the N/P ratio lower. To our knowledge, no other study has analyzed the influence of Si on root stoichiometry of wetland graminoids, even though root biomass is an important part of total biomass and is responsible for most peat formation in fen peatlands [5] and, thus, it should be investigated individually [21].

FTIR ratios only allow interpretation of relative shares of compounds, not absolute contents (Table 1). Nevertheless, the original *Eriophorum* shoots showed decreasing FTIR ratios with an increasing Si mass fraction of the shoots, for both, the “low-P” and the “high-P” treatment group (Table A3). The share of recalcitrant material, hence, decreases with increasing Si content. In line with our findings, also others [11,17] found a negative correlation between Si content and lignin content of rice straw. Schoelynck et al. [22] used extraction methods to determine the cellulose and lignin content of wetland plants. In their study, for wetland graminoids like *Phragmites australis* and *Glyceria maxima,* they found no relationship between lignin and Si content in the plants. Schaller et al. [23], however, found decreasing cellulose/silicon and lignin/silicon ratios in *Phragmites australis* with increasing Si availability when using extraction techniques. Here, the roots of *Eriophorum* showed decreasing FTIR ratios with an increasing Si mass fraction of the shoots for the “low-P” treatment group. For the “high-P” treatment group only small differences could be observed, while a decreasing trend with an increasing Si mass fraction was still discernible.

This change of compound share under higher availability of Si was expected since graminoids can replace structures like lignin, aromatic, carbonylic, carboxylic and aliphatic structures by precipitation of Si, as Schaller et al. [11] demonstrated for rice plants. These authors also found a relative reduction in the share of wax and lipids, free organic acids and lignin with increasing Si content [11,23]. The effect of Si on *Eriophorum* shoot structural C composition was overall larger for shoots than for roots. The roots only showed a strong change in structural C composition in the low-P treatment group. Si is primarily transported to the shoots instead of the roots [24], which might be a reason for the clearer Si effect of structural C composition in the shoots compared to the roots.

### 3.2. Decomposition

Upon investigating the decomposition of the *Eriophorum* shoots, we hypothesized (II) that litter with a high Si mass fraction would show higher CH_4_ and CO_2_ production rates due to lower shares of recalcitrant organic matter, such as phenols and aliphatic groups. To this end, we analyzed the time course of CO_2_ and CH_4_ production rates. Only during the initial phase (day 1–11), did Si seem to increase the CO_2_ production rate. In relation to the entire incubation (1–72), there was more CO_2_ produced in the Si 0% treatments, compared to the other treatments. This difference was, thus, primarily due to differences in the initial phase. This finding contradicts the findings of Schaller and Struyf [12], who found higher decay rates of litter with increasing Si content of the litter, even though our experiment also had a lower share of phenols in the material.

The result of our experiment contradicted our expectation: Higher CO_2_ production was found in treatments with a very low Si mass fraction, and lower CO_2_ production rates were found in treatments with shoots which had a high Si mass fraction. In the long-term incubation phase, there was no significant difference between the treatments, though, except the peat control treatment, which showed much lower rates than the treatments with litter addition. In accordance with Gao et al. [25], CO_2_ production was higher in the beginning of the experiment compared to the end of the experiment. According to Hömberg et al. [26], we expected increased CO_2_ production in the treatments that had received Si-rich plant material. The differences between both experiments were either due to the different ways in which Si was added - either as inorganic salt (Na_2_O_3_Si) or as a plant bound biogenic Si (this study). Another possibility would be that this difference was caused by Si interacting with other plant constituents like N, P, S, and the C quality, yet we cannot draw conclusions from data available here.

The differences in CH_4_ production rates agreed, to a large extent, with our hypothesis. We expected greater CH_4_ production in the high-Si treatment compared to the no-Si treatment. These differences, while not significant due to high variability, showed clear trends. Reithmaier et al. [14] also found increased CH_4_ concentrations in their field study, where peat was amended with amorphous Si. Our finding is also in accordance with the results from Hömberg et al. [26], even though both experiments did not use Si bound in biomass but used amorphous SiO_2_ or Na_2_O_3_Si added as inorganic fertilizer. The low CH_4_ production during the initial phase of the incubation coincides with a high share of phenols in the low-Si shoot material, especially in the low-P treatments. High concentrations of phenols have been shown to inhibit methanogenic processes [27,28].

The water phase showed an increase in Si and Fe concentrations over the time of the incubation, whereas the concentrations of TOC and P decreased. The increase in Si may be explained by progressive leaching of Si from the shoot material during decomposition. Like Schaller and Struyf [12], we found that from material with higher initial Si mass fractions, more Si was leached. Yet, the increase in leached Si was not proportional to the increase in the mass fraction of Si, which was probably due to the higher CO_2_ production and thus C mineralization rate in the “Si 0%” treatment, potentially releasing comparatively more Si. The decrease in DOC, was pronounced for the “Si 0%” treatment, whereas the decrease in dissolved P was most intense for the “Si 10%” treatment. The decrease in DOC, especially from those shoots with a low Si mass fraction, can be attributed to the high degradability of C leached from *Eriophorum* shoots [29] and the correspondingly higher CO_2_ production rate. The decrease in P concentration was pronounced for the shoots with a high Si mass fraction, even though this was not due to the P mass fraction in the shoots, which showed no differences. Probably, there was higher P leaching from those shoots that contained more Si. This could be due to extremely fast release of Si from graminoid shoots [30], and thereby, release of easily utilizable P structures [31,32,33]. It may also be explained by the competition of silicic acid and P regarding their binding to soil particles [26,32]. The binding affinity of silicic acid to soil particles is depending on its speciation. Polysilicic acid, which is the predominant species during dissolution of amorphous Si, shows particularly high binding affinity [34]. Schaller and Struyf [12] found the water phase of their high-Si incubation to contain lower concentrations of P during the experiment, which was not the case in our experiment (higher P concentrations in the Si 10% treatment compared to the Si 0% treatment).

The C/N ratio in the water phase decreased between the initial phase and the end of the incubation due to decreasing C concentrations, but there were no differences between the treatments concerning this decrease in the water phase. The C/N ratio of the *Eriophorum* shoots generally decreased as well during the decomposition period. In the “low-P” treatment group, the decrease was more pronounced for the Si addition treatments, compared to the treatment without Si addition. The C/N ratio decreased due to higher absolute losses in the C mass fraction compared to losses in the N mass fraction. The N mass fraction was higher in those treatments that did not receive Si fertilization. Matzner and Berg [35] found high N contents in litter to act as trigger for decomposition, whereas it inhibits decomposition in later stages of decomposition. The comparatively high CO_2_ production rate in the Si 0% treatments can also be interpreted as triggered by high N mass fractions of the litter. The “low-P Si 10%” treatment showed an increase in the C mass fraction after the incubation. The mass fraction in % always refers to the total material measured. The increase in C mass fraction, here, is explained by a strong leaching of Si from the plant material of this treatment. The share of C in this material, thus, increased during the incubation. In all other treatments the Si leaching was smaller and the release of C into the solution was higher.

We further hypothesized (III) the onset of methanogenesis to be earlier for the decomposition of Si-rich litter, than for Si-poor litter. We expected this due to several processes. First the available electron acceptors should be depleted more rapidly due to the higher CO_2_ production, which we expected (hypothesis II) [14]. Second, we expected a generally higher CH_4_ production due to a direct stimulating effect of Si. We confirmed this hypothesis (III) for the *Eriophorum* shoot incubation: The onset of methanogenesis for the Si 10% treatment was earlier than that of the Si 0% treatment, such that more CH_4_ was produced during the experiment in these incubations. Until methanogenesis began, unexpectedly less CO_2_ was produced in the Si-addition treatments than in the treatments without Si. The electron acceptors in the Si-addition treatments had thus been used up more rapidly and apparently incompletely, since methanogenesis started earlier despite lower CO_2_ production before onset of methanogenesis. Although this suggests another direct effect of Si addition on methanogenesis or pathways of anaerobic C mineralization, we cannot clarify this aspect from available data of this experiment.

This partly met our expectation of the hypothesis (I). However, the reasons for the different behavior of CO_2_ and CH_4_ production rates remain unclear. As discussed before, the high-Si treatment did not show a higher CO_2_ production rate. During long-term incubation, there was no difference in CO_2_ production rates, and during the initial phase, the Si 0% treatment showed the highest production rate.

Wainwright et al. [13] found Si to have a positive effect on the growth of fungi in both oligotrophic and nutrient rich media, and Voronin and Mukhin [36] found saprotrophic fungi to be an initial key factor to feed anaerobic degradation pathways ending up in methanogenesis. We can only speculate about the abundance of fungi and methanogens in our experiments, but higher abundances of fungi, which initiate the decomposition of lignin and cellulose for further fermentation and oxidation and the concomitant high abundance of methanogens, as described by Voronin and Mukhin [36], could be a reason for the high CH_4_ production rates we observed when Si availability was high.

In relation to the litter quality assessed by FTIR, the share of phenolic compounds increased strongly during the incubation for the Si 1% and the Si 10% treatment of the low-P treatment group, and it increased for the Si 0% and the Si 10% treatment of the high-P treatment group. The increase in the share of phenolic OH in the “low-P Si 10%” was highest compared to the “low-P Si 1%” and the “low-P Si 0%” treatment. For the high-P treatment group the magnitude of the increase did not differ with the Si treatment. The strong increase in the share of phenolic OH in the remaining litter of the “low-P Si 10%” treatment coincided with a high CH_4_ production rate, but also with a comparatively low CO_2_ production rate.

It is likely that microbial communities developed differently in the treatments due to the experimental amendments. This was, however, beyond the scope of our study. Nevertheless, we pretreated all incubations in the same way by inoculating them with the same peat solution. Therefore, the different incubations are as comparable as possible.

When graminoid litter with different Si mass fractions undergoes decomposition in anaerobic environments like fen ecosystems, differences in the Si mass fraction and the simultaneous changes obviously influence the decomposition and the decomposition pathways of the litter, as shown in our study. Nevertheless, the CO_2_ production rate in the decomposition of *Eriophorum* shoots was not influenced by the Si mass fraction of the litter, even though Si changed the stoichiometric composition of the biomass. Albeit not significantly, the CH_4_ production rate was apparently higher for litter with a higher Si mass fraction.

Therefore, different input rates of plant available Si—whether from bedrock weathering or from external sources (ash, precipitation, runoff from the catchment) - may influence the decomposition processes of the vegetation grown under such different Si availabilities, and thus, affect greenhouse gas production in peatlands by stimulating higher and more rapid CH_4_ production under high Si availability.

The roots, the main peat-forming parts of the graminoid vegetation, are not expected to be influenced by different Si availabilities directly, since different Si availabilities in the soil do not necessarily lead to significantly higher Si mass fractions in the roots. The lack of root aphids in the Si 10% treatment of the first part of the study, however, suggests that Si may have an indirect influence on root growth, by inhibiting pest infestation. Overall, Si was accumulated rather in the shoots than in the roots. The shoots that grew under high Si availability, however, accumulated Si according to its availability. When this shoot material decomposes, it decomposes faster than the roots and is the main contributor to CO_2_ and CH_4_ emissions from a fen peatland [21]. The Si effect on CO_2_ production rates was fast, leading to higher CO_2_ production from the shoots that grew under low Si availability when the plants got in contact with water, e.g., in water filled depressions or hollows. The CH_4_ effect was delayed and displayed, in contrast, higher CH_4_ production from the shoots grown under high Si availability.

The root biomass may be less prone to diseases or pests and the overall productivity may increase if the Si input into an ecosystem increases, but this remains to be tested. The changes in stoichiometry and carbon compounds only have a small influence on CO_2_ production, but CH_4_ production from decaying shoot biomass may increase with increasing Si availability.

## 4. Methods

### 4.1. Part I—Biomass Characterization

#### 4.1.1. Extraction and Preparation of Plants

The investigated plant species *Eriophorum vaginatum*, L. was chosen for this experiment due to its broad abundance in minerotrophic peatlands [5]. The planting experiment was also conducted with *Carex rostrata* Stokes, but it showed only poor growth within the experimental period, and hence, the results are presented in the supporting material only.

Individual plants were sampled in autumn 2018 from the minerotrophic peatland “Schlöppnerbrunnen” in the German Fichtelgebirge mountain range (geographic coordinates: 50°7′56.83″ N; 11°52′53.94″ E) at 706 m a.s.l. This research site was formerly used by many other authors [37,38,39]. The sampling was done using a spade and a saw to get intact peat sods and preserve a root length of at least 20–30 cm. The material was transported in plastic boxes to the University of Bayreuth and stored in a cold greenhouse. After excavation, individual plants were separated and washed to remove adherent peat. The root length and the length of aboveground biomass was adjusted for the 30 individuals to 10 cm long roots and 5 cm long shoots. The individuals were stored with their roots immersed in water over night.

#### 4.1.2. Substrate Preparation

The substrate for the planting experiment was based on unamended horticultural fibric peat from a peatland in Lower Saxony, Germany. This peat was, depending on the treatment, mixed with different portions of SiO_2_ (added as fumed silica, Aerosil-300, Evonik, Germany). The mixing was done in a concrete mixer for approximately 20 min. The control treatment contained no added SiO_2_ and had a background mass fraction of 0.16% (±0.03) Si as measured by X-ray fluorescence (XRF) (procedure described in chapter 4.3). It was likewise pre-treated in the concrete mixer to minimize differences, due to handling. The Si 1% treatment contained 1% weight SiO_2_ and the Si 10% treatment contained 10% SiO_2_, resulting in three types of substrates. 500 g of substrate was filled in 20 pots each. The substrate was moistened with 1 L of deionized water per pot.

#### 4.1.3. Planting and Fertilization

Two days after sampling in the field, the plants were planted in the prepared pots. Each species was planted in each substrate type in 10 replicates.

To favor plant growth, the plants were fertilized three times (altogether 100 mL) during the growing season. The fertilizer was prepared with two different amounts of PO_4_ to establish two different P treatments (Table 2). The first fertilization was applied directly after planting (resulting in 2.9 mg P, and 17.3 mg P per pot respectively). Five pots of each plant and substrate type received the low-P fertilizer, whereas the others received the high-P fertilizer. The second fertilization was applied after one month, the third after three months with a smaller amount of fertilizer on both occasions (resulting in 2.2 mg P and 13,0 mg P per pot respectively) (see also Table A1 in Appendix A). This resulted in six different treatments per species, three levels of Si in the substrate and two levels of P in the fertilization in a full factorial design.

The experiment was conducted in a greenhouse at a temperature of 20 °C and with greenhouse lights (400 W, 35,000 lm, Powerstar HQI-TS 400W D Pro, Osram, Munich, Germany) and a diurnal light cycle based on natural conditions of late spring in the mid-latitudes (14 h light, 10 h dark). The pots were watered every second day with distilled water to avoid application of further nutrients. The pore water was regularly checked for changes in pH.

#### 4.1.4. Harvest, Milling

With the start of senescence, watering was stopped two weeks before harvesting the plants to facilitate senescence. The aboveground biomass was clipped and stored in paper bags. Thereafter, the substrate was removed manually from the roots first by shaking off most parts of the dry soil and then cleaning the roots from the adhered peat under constantly flowing water; the roots were also then stored in paper bags. One additional paper bag was taken per treatment to collect a pooled sample of material of plant species of each treatment and litter type. This material was later used for the incubation experiment (see below). After harvesting the above- and belowground material, the biomass in all paper bags was dried in a drying cabinet at about 20 °C for one week. The low temperature was chosen to maintain environmental conditions.

Each individual plant sample (~120 in total) was ground using an ultra-centrifugal mill (Retsch, ZM1, Haan, Germany) at 10,000 rpm. This material was used for P and Si extractions and for C and N elemental analysis.

#### 4.1.5. Analysis of the Biomass

All samples were analyzed on their Si, P, N and C mass fraction. The mass fraction of amorphous Si was determined by alkaline extraction following the procedure of Meunier et al. [40]. 30 mg of the milled material was funneled into 50 mL flat bottom tubes. Then, 30 mL of 0.1 M Na_2_CO_3_ (Roth) solution was added, the tubes were shaken, closed with lids, and then boiled for 5 h at 85 °C in a heating block (DigiPrep Jr, SCP Science, Montreal, Canada). The tubes were shaken again once each hour. After the boiling period, we waited for the solid parts to settle before filtering the supernatant through 0.2 µm PET syringe filters (Macherey-Nagel, Düren, Germany). The filtered supernatant was retained in polypropylene tubes. The concentration of total Si was then analyzed with an ICP OES (Varian, Vista-pro radial, Palo Alto, USA) in the central analytical laboratory of BayCEER, Bayreuth, Germany. The total P content was determined following EN 13805, 2002 [41]. Prior to sample digestion, microwave vessels were cleaned by a blank digestion. Afterwards, 150–250 mg of the ground plant material was added to the microwave tubes. A total of 3 mL of 65% HNO_3_ (Roth) and 2 mL of 30% H_2_O_2_ were added, and the tubes were closed with lids and shaken. After 14 h of resting, the tubes were heated in a microwave to 180 °C for 20 min and subsequently kept boiling for 15 min. After cooling down, the tubes were opened and quantitatively transferred into new vials with ultra-pure water. Therefore, a diluted sample of 50 mL was gained. The P mass fraction of these samples was determined again by ICP OES (Varian, Vista-pro radial, Palo Alto, USA) as above. The C and N mass fractions of the milled material were determined with a CN elemental analyzer (EA 3000, Eurovector/Hekatech, Milano, Italy/Wegberg, Germany). Therefore, approximately 3 mg of the dried and milled plant material was balanced and funneled in a tin capsule and subsequently analyzed at the Institute of Landscape Ecology at the University of Münster, Germany.

#### 4.1.6. FTIR Analysis

The pooled plant biomass samples were stored in the dark and transported to the University of Münster. Here, the material was cut into approximately 1 cm long pieces by hand, thoroughly mixed and again stored dry and in the dark until further use.

A subsample of the pooled plant material was finely ground using a mixer mill (Retsch MM 400). Then, 2 mg of the finely ground sample were mixed with 200 mg of potassium bromide (KBr, IR grade, Sigma Aldrich, St. Louis, UAS) in a mortar, obtaining a homogenous powder. The powder was placed in a pelleting press and pressed into 13 mm pellets at a load of 8 t. The pellets were immediately transferred into the FTIR spectrometer (Cary 600, Agilent, Santa Clara, CA, USA), and 32 scans of the sample were recorded and averaged to obtain the final infrared absorption spectrum. The spectra were preprocessed in R [42] using the function ir_bc() from the R package “ir” [43] (version 0.0.0.9000) which is based on a “rubberband” algorithm from the spc.hyperspec() function of the R package hyperSpec [44]. To interpret the FTIR spectra, we assigned absorption features to major structural moieties in organic matter as explained in Table 1.

### 4.2. Part II—Plant Decomposition Experiment

#### 4.2.1. Preparation of the Incubation

The incubation experiment with *Eriophorum* shoots was performed in 120 mL injection bottles (Glasgerätebau Ochs, Lenglern, Germany). For the incubation, 500 mg of dry plant material was funneled into the bottles. Then 200 mg of dry peat was added. The peat used here was a long-term oxidized sod peat from a bog in Lower Saxony, Germany (see Agethen and Knorr [7] for characterization of this peat material). It was crumbled by hand and then sieved through a 2 mm sieve to obtain homogenous material. Subsequently, 30 mL of ultrapure water was added. To inoculate the incubations with an active microbial community of a fen, 100 µL of fen peat filtrate (approximately 30 µm pore size) from the Schlöppnerbrunnen fen was added.

The bottles were closed with butyl rubber stoppers and then flushed with nitrogen for about 20 min. Thereby, oxygen (O_2_) and CO_2_ in the bottles were removed and the bottles prepared for the following incubations. This resulted in six treatments with *Eriophorum* shoots (see variants of biomass above) and a control treatment without plant material (Table A2).

#### 4.2.2. Incubation Procedure

During the entire experiment, the bottles were stored in an incubator at 15 °C in the dark, and they were only shortly removed for samplings. In regular intervals, measurements of the CO_2_ and CH_4_ concentration in the headspace of the incubation bottles were taken using a gas chromatograph (SRI Instruments, Earl St. Torrance, USA) equipped with an FID detector and methanizer. An amount of 2 mL of N_2_ was injected into the headspace, mixed with the headspace gas, and then 2 mL of diluted gas were taken from the bottles. This was done to avoid a change of pressure in the headspace over longer time periods. The fist GC measurement in the shoot incubation was performed 24 h after the N_2_ flushing. It was followed by three more measurements within 10 days; this period is hereafter referred to as “initial phase”. Thereafter, the bottles were opened, 4 mL of water was taken with a syringe and a cannula and immediately filtered through 0.22 µm nylon syringe filters (MACHEREY-NAGEL, Düren, Germany) into centrifuge tubes. Then, 10 mL of ultrapure water was added to the incubation bottles to ensure to have enough water for a second sampling. This was followed by 20 s of intense N_2_ flushing, closure again with butyl rubber stoppers, and 20 min N_2_ flushing of the headspace. Twenty-four hours after flushing, the next concentration measurement was taken at the GC. From then on, these measurements were conducted for 13 weeks with measurements once a week, in order to capture further ongoing decomposition after initial leaching and decomposition effects had passed. This was the “long-term phase” of the incubation.

The water samples from the incubations were prepared for different analyses: The concentrations of dissolved organic carbon (DOC) and dissolved nitrogen (TN) were measured using a V-CPN Analyzer, (Shimadzu, Tokyo, Japan). To determine major anions, ion chromatography (883 Basic IC plus Metrohm, Herisau, Switzerland) was employed, and for the total concentration of other major elements such as Si and total P, we used inductively coupled plasma optical emission spectroscopy (ICP-OES; SpectroBlue, Kleve, Germany).

#### 4.2.3. Analysis after Decomposition

After the 14 weeks of incubation, water sampling was done directly after opening the bottles. The samples were treated and analyzed in the same way as described above. Then, the still discernible plant fragments were collected from the bottles, freeze dried (Christ Alpha 1-4 LDplus, Osterode am Harz, Germany) and milled with the same equipment as used on the initial material. Afterwards, the material was characterized again using FTIR spectroscopy (see above).

#### 4.2.4. Statistical Analysis

Calculations on statistically significant differences were done using R (R-Core-Team, 2018). We assumed a Gaussian distribution within the treatment groups. After a test on homoscedasticity (var test) a *t*-test (*t* test) or a *welch*-test (*t* test) was performed, respectively, depending on the results of the prior test. The significance level was set to *α* = 0.05. Diagrams were created using R [42] and the R package Hmisc [45]. Unless otherwise noted, the standard deviation of the mean is given in bar plots.

### 4.3. Wild Plant Characterization for Comparison

In summer 2018, 24 shoot samples were collected from plants of three species from the Schlöppnerbrunnen fen, the same site at which the plants for the growing experiment were taken. These species were *Carex rostrata*, *Eriophorum vaginatum* and *Molinia caerulea* Moench. The shoots were frozen and subsequently freeze dried (Christ Alpha 1-4 LDplus, Osterode am Harz, Germany).

The plants were milled according to the procedure described in Section 4.1.4. Milled samples were subjected to an FTIR analysis (as described in Section 4.1.6) and total element mass fractions were determined with X-ray fluorescence spectroscopy at the University of Münster, Germany. For the latter, a wavelength dispersive X-ray fluorescence spectrometer (WD-XRF Rigaku ZSX Primus II, Tokyo, Japan) was used. Therefore, 500 mg of the sample was formed into a pellet at a load of 6–7 t using a pellet die (Specac, Orpington, UK), and pellets were stored in polyethylene bags in a desiccator until measurement.

## 5. Conclusions

Do higher Si mass fractions go along with decreased mass fraction of nutrients and changes in shares of carbon compounds (decrease of hardly degradable compounds)? This was found for the N mass fraction of Eriophorum, especially for the shoots. The FTIR ratios displayed a decreasing share of lignin, aromatic C=C and COO^−^ carbonylic and carboxylic, aliphatic structures (waxes and lipids) and phenolic OH with increasing an Si mass fraction. The roots accumulated much less than Si. Since the roots are the main peat forming tissues, they are obviously less influenced by different Si availabilities than the shoots.

Does the Si mass fraction and the concomitant changes (nutrient mass fraction, share of carbon compounds) influence the decomposition of plant litter from fens? For Eriophorum shoots, the CH_4_ production rate was apparently higher, albeit not significantly, for litter with a higher Si mass fraction. The CO_2_ production rate was only during the initial phase influenced by the Si mass fraction. It displayed a lower CO_2_ production rate for higher Si mass fractions. Afterwards, the CO_2_ production was equal between the treatments. The influence of high Si mass fractions on decomposition processes, thus, differs between the respective pathway and probably also with species and with tissue.

Is litter grown under high Si uptake less recalcitrant than litter grown under low Si mass fractions? The share of recalcitrant compounds seems to be higher in those plants that have a low Si mass fraction. This does not seem to influence CO_2_ production rates in the long-term. Meanwhile, the CH_4_ production tends to be faster and set earlier in incubations with litter having an increasing Si mass fraction, and with an altered share of different carbon compounds.

## Figures and Tables

**Table 1:** Wavenumbers used for FTIR ratios, their indication and references.

Wavelength or Ratio	Indicative of	Reference
1090	Polysaccharides	Broder et al. [15]
1350/1090	symmetric -COO^−^ stretch and/or -CH bending of aliphatic structures	Niemeyer et al. [16]
1630/1090	Aromatic C = C and COO^−^ // aromatics and aromatic or aliphatic carboxylates	Broder et al. [15]
1720/1090	Aromatic C = C or C = O of amides	Broder et al. [15]
2850/1090	Symmetric CH2, aliphatic structures, waxes, and lipids	Schaller et al. [11] Agethen and Knorr [7]
2920/1090	Antisymmetric CH2, waxes and lipids	Agethen and Knorr [7] Schaller et al. [11]
3380/1090	OH^−^ stretch of phenolic structures	Niemeyer et al. [16]

**Table 2:** Composition of the fertilizer solution added per pot over the complete growing season. Two different fertilizers were prepared, one containing a small concentration of K_2_HPO_4_, the other one containing a high concentration of K_2_HPO_4_.

Nutrient	K	P	N	Ca	Cl	Mg	B	Mo	Na	Zn	Cu	Co	Mn	Fe	S
**mg/100 mL** **(≙ mg per pot)**	18.1 *or* 109.5	7.1 *or* 43.4	28.0	3.4	3.6	4.9	0.26	0.9	6.4	1.43	0.13	0.04	0.98	0.5	7.6

**Figure A3:**
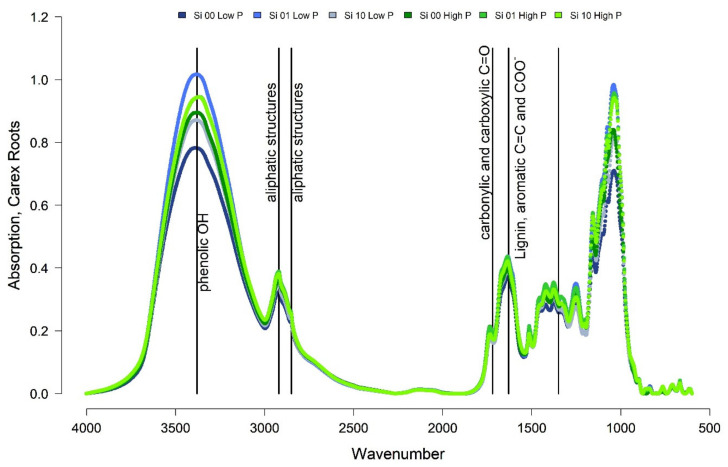
FTIR Spectra of *Carex* roots before the incubation. Low P fertilized plants are shown in blue, high P fertilized plants are shown in green.

## Data Availability

Data is contained within the article.

## Appendix A

**Table A1:** Composition of fertilizer solutions 1–5 in the applied concentration.

Fertilizer Number	Nutrient	mmol L^−1^ in Low-P Fertilizer	mmol L^−1^ in High-P Fertilizer
**1**	K_2_HPO_4_	*400.6*	*2438.4*
	NH_4_NO_3_	800.4	800.4
**2**	CaCl·2H_2_O	62.7	62.7
**3**	MgSO_4_	500.3	500.3
**4**	H_3_BO_3_	14.8	14.8
	NaMoO_4_·2H_2_O	24.2	24.2
**5**	ZnSO_4_·7H_2_O	63.3	63.3
	CuSO_4_·5H_2_O	5.0	5.0
	CoSO_4_·7H_2_O	2.0	2.0
	MnCl·4H_2_O	29.2	29.2
	FeSO_4_·7H_2_O	25.0	25.0
	Na_2_EDTA·2H_2_O	498.8	498.8

**Table A2:** Treatment additions in the decomposition experiments.

Treatment	Shoots	Peat for *Eriophorum*- Shoot Incubation
Low-P; Si 0%	500 mg	200 mg
Low-P; Si 1%	500 mg	200 mg
Low-P; Si 10%	500 mg	200 mg
High-P; Si 0%	500 mg	200 mg
High-P; Si 1%	500 mg	200 mg
High-P; Si 10%	500 mg	200 mg
Peat control	/	200 mg

The peat + biomass treatments and the peat control were 5 times replicated. All treatments received 30 ml of ultrapure water and an inoculum of 100 µL fen DOM filtrate.

**Table A3:** Selected FTIR ratios of plants before and after incubation.

	1350/1090	1630/1090	1720/1090	2850/1090	2920/1090	3380/1090
***Eriophorum*** **shoots before incubation**						
Si 0%	0.493	0.572	0.372	0.466	0.663	1.263
Si 1% low P	0.441	0.509	0.343	0.395	0.570	1.122
Si 10% low P	0.373	0.415	0.284	0.302	0.441	0.889
Si 1% high P	0.428	0.442	0.321	0.374	0.539	1.076
Si 10% high P	0.362	0.396	0.274	0.288	0.425	0.866
***Eriophorum*** **shoots after incubation**						
Si 0% low P	0.511	0.593	0.487	0.484	0.675	1.092
Si 1% low P	0.448	0.521	0.277	0.394	0.556	1.319
Si 10% low P	0.463	0.522	0.278	0.411	0.573	1.332
Si 0% high P	0.428	0.453	0.248	0.380	0.539	1.303
Si 1% high P	0.465	0.511	0.427	0.419	0.585	0.968
Si 10% high P	0.393	0.449	0.352	0.346	0.498	0.931
***Eriophorum*** **roots before incubation**						
Si 0% low P	0.525	0.633	0.504	0.501	0.688	1.207
Si 1% low P	0.482	0.520	0.438	0.441	0.617	1.096
Si 10% low P	0.396	0.422	0.343	0.340	0.485	0.882
Si 0% high P	0.433	0.525	0.275	0.367	0.524	1.279
Si 1% high P	0.446	0.511	0.283	0.377	0.533	1.265
Si 10% high P	0.406	0.450	0.230	0.347	0.506	1.297
***Carex*** **roots before incubation**						
Si 0% low P	0.5075	0.68918	0.3124	0.4237	0.6045	1.4629
Si 1% low P	0.4519	0.5942	0.24698	0.3380	0.5157	1.3947
Si 10% low P	0.4340	0.6517	0.2708	0.3919	0.5796	1.4432
Si 0% high P	0.4810	0.6138	0.3014	0.3995	0.5706	1.3940
Si 1% high P	0.4490	0.6030	0.2749	0.3675	0.5486	1.3296
Si 10% high P	0.4186	0.6001	0.2521	0.3683	0.5518	1.3658

**Table A4:** Tables of *p*-values belonging to Figure 6.

**Si**	**Time**	**Low P Si 0%**	**Low P Si 0%**	**Low P Si 1%**	**Low P Si 1%**	**Low P Si 10%**	**Low P Si 10%**	**High P Si 0%**	**High P Si 0%**	**High P Si 1%**	**High P Si 1%**	**High Si 10%**	**High Si 10%**	**Peat Control**	**Peat Control**
**Time**	**/**	**Beg.**	**End**	**Beg.**	**End**	**Beg.**	**End**	**Beg.**	**End**	**Beg.**	**End**	**Beg.**	**End**	**Beg.**	**End**
**Low P Si 0%**	**Beg.**	/	5.364 × 10^−9^	0.0079	*2.361* × *10^−6^*	0.0079	*0.0098*	0.5476	4.549 × 10^−8^	0.0079	*1.661* × *10^−6^*	0.0079	*8.786* × *10^−6^*	0.0079	4.029 × 10^−7^
**Low P Si 0%**	**End**		/	/	0.0079	/	0.0357	/	0.0714	/	0.0079	/	0.0079	/	0.0079
**Low P Si 1%**	**Beg.**			/	*3.704* × *10^−6^*	0.0079	*0.0114*	0.0079	4.765 × 10^−7^	0.0925	*2.596* × *10^−6^*	0.0079	*1.166* × *10^−5^*	0.0079	0.2764
**Low P Si 1%**	**End**				/	/	0.0357	/	0.0357	/	0.6905	/	0.0079	/	0.0079
**Low P Si 10%**	**Beg.**					/	*0.0138*	0.0079	0.0056	0.0079	1.638 × 10^−7^	0.8413	3.995 × 10^−8^	0.0079	*0.0012*
**Low P Si 10%**	**End**						/	/	0.0182	/	*0.0357*	/	1	/	0.0357
**High P Si 0%**	**Beg.**							/	7.093 × 10^−7^	0.0079	2.408 × 10^−9^	0.0079	*4.064* × *10^−6^*	0.8413	*0.0003*
**High P Si 0%**	**End**								/	/	0.0357	/	0.0357	/	0.0357
**High P Si 1%**	**Beg.**									/	*4.547* × *10^−6^*	0.0079	*1.294* × *10^−5^*	0.0079	0.0016
**High P Si 1%**	**End**										/	/	0.0079	/	0.0079
**High P Si 10%**	**Beg.**											/	*2.331* × *10^−5^*	0.0079	1.797 × 10^−8^
**High P Si 10%**	**End**												/	/	0.0079
**Peat Control**	**Beg.**													/	*2.521* × *10^−7^*
**Peat Control**	**End**														/
**P**	**Time**	**Low P Si 0%**	**Low P Si 0%**	**Low P Si 1%**	**Low P Si 1%**	**Low P Si 10%**	**Low P Si 10%**	**High P Si 0%**	**High P Si 0%**	**High P Si 1%**	**High P Si 1%**	**High Si 10%**	**High Si 10%**	**Peat Control**	**Peat Control**
**Time**	**/**	**Beg.**	**End**	**Beg.**	**End**	**Beg.**	**End**	**Beg.**	**End**	**Beg.**	**End**	**Beg.**	**End**	**Beg.**	**End**
**Low P Si 0%**	**Beg.**	/	0.2391	0.1439	0.0041	0.0004	0.2017	2.264 × 10^−6^	0.0005	3.784 × 10^−8^	4.255 × 10^−6^	7.262 × 10^−8^	4.923 × 10^−6^	*7.918* × *10^−5^*	*5.355* × *10^−5^*
**Low P Si 0%**	**End**		/	/	0.0130	/	0.0303	/	7.772 × 10^−5^	/	3.711 × 10^−7^	/	1.483 × 10^−6^	/	*2.116* × *10^−5^*
**Low P Si 1%**	**Beg.**			/	0.0384	2.491 × 10^−5^	0.022	2.381 × 10^−7^	7.704 × 10^−5^	4.039 × 10^−9^	3.837 × 10^−7^	2.29 × 10^−8^	1.372 × 10^−6^	*5.248* × *10^−5^*	3.143 × 10^−5^
**Low P Si 1%**	**End**				/	/	0.0010	/	3.48 × 10^−6^	/	7.562 × 10^−9^	/	4.758 × 10^−8^	/	5.55 × 10^−7^
**Low P Si 10%**	**Beg.**					/	0.0144	6.628 × 10^−5^	0.0341	8.207 × 10^−8^	0.0002	2.486 × 10^−7^	7.605 × 10^−5^	*4.431* × *10^−6^*	*2.576* × *10^−6^*
**Low P Si 10%**	**End**						/	/	0.0100	/	0.0712	/	0.0061	/	0.0013
**High P Si 0%**	**Beg.**							/	0.0159	0.0003	0.1461	3.799 × 10^−6^	0.0254	*1.063* × *10^−6^*	*5.663* × *10^−7^*
**High P Si 0%**	**End**								/	/	0.0712	/	0.0061	/	0.0013
**High P Si 1%**	**Beg.**									/	1.409 × 10^−6^	*0.0003*	*0.1128*	*7.13* × *10^−8^*	*1.599* × *10^−8^*
**High P Si 1%**	**End**										/	/	0.0041	/	4.54 × 10^−7^
**High P Si 10%**	**Beg.**											/	0.0005	*3.144* × *10^−6^*	*2.541* × *10^−6^*
**High P Si 10%**	**End**												/	/	1.361 × 10^−5^
**Peat Control**	**Beg.**													/	*0.0001*
**TOC**	**Time**	**Low P Si 0%**	**Low P Si 0%**	**Low P Si 1%**	**Low P Si 1%**	**Low P Si 10%**	**Low P Si 10%**	**High P Si 0%**	**High P Si 0%**	**High P Si 1%**	**High P Si 1%**	**High Si 10%**	**High Si 10%**	**Peat Control**	**Peat Control**
**Time**	**/**	**Beg.**	**End**	**Beg.**	**End**	**Beg.**	**End**	**Beg.**	**End**	**Beg.**	**End**	**Beg.**	**End**	**Beg.**	**End**
**Low P Si 0%**	**Beg.**	/	*0.0036*	8.157 × 10^−7^	1.676 × 10^−5^	0.0002	4.849 × 10^−7^	0.0521	5.752 × 10^−7^	0.0023	*0.0020*	0.0005	6.707 × 10^−8^	*2.631* × *10^−7^*	*1.614* × *10^−7^*
**Low P Si 0%**	**End**		/	/	0.1042	/	0.5271	/	*0.2313*	/	0.3314	/	*0.3161*	/	*0.0002*
**Low P Si 1%**	**Beg.**			/	*0.0076*	*0.0230*	0.0014	*0.0007*	0.0664	7.158 × 10^−5^	*0.0395*	*0.6043*	3.678 × 10^−6^	*7.508* × *10^−9^*	6.019 × 10^−15^
**Low P Si 1%**	**End**				/	/	0.09877	/	*0.0100*	/	0.5478	/	*0.2217*	/	*0.0002*
**Low P Si 10%**	**Beg.**					/	*6.669* × *10^−5^*	0.0002	0.0030	0.0382	*0.0130*	0.0850	9.045 × 10^−6^	*1.085* × *10^−6^*	*7.892* × *10^−7^*
**Low P Si 10%**	**End**						/	/	0.0004	/	*0.5011*	/	0.3025	/	*2.692* × 10^−6^
**High P Si 0%**	**Beg.**							/	*0.0005*	0.0012	0.0001	0.0002	1.158 × 10^−6^	*4.445* × *10^−6^*	3.984 × 10^−6^
**High P Si 0%**	**End**								/	/	*0.0538*	/	1.27 × 10^−5^	/	*2.169* × *10^−9^*
**High P Si 1%**	**Beg.**									/	0.0012	0.0092	7.252 × 10^−7^	*4.377* × *10^−7^*	2.795 × 10^−7^
**High P Si 1%**	**End**										/	/	*0.0538*	/	*1.27* × *10^−5^*
**High P Si 10%**	**Beg.**											/	0.0105	*3.64* × *10^−5^*	3.326 × 10^−5^
**High P Si 10%**	**End**												/	/	*2.169* × *10^−9^*
**Peat Control**	**Beg.**													/	*0.0072*
**Peat Control**	**End**														/
**Fe**	**Time**	**Low P Si 0%**	**Low P Si 0%**	**Low P Si 1%**	**Low P Si 1%**	**Low P Si 10%**	**Low P Si 10%**	**High P Si 0%**	**High P Si 0%**	**High P Si 1%**	**High P Si 1%**	**High Si 10%**	**High Si 10%**	**Peat Control**	**Peat Control**
**Time**	**/**	**Beg.**	**End**	**Beg.**	**End**	**Beg.**	**End**	**Beg.**	**End**	**Beg.**	**End**	**Beg.**	**End**	**Beg.**	**End**
**Low P Si 0%**	**Beg.**	/	2.224 × 10^−7^	0.1022	4.575 × 10^−6^	7.101 × 10^−6^	0.0102	0.0543	1.448 × 10^−5^	3.548 × 10^−5^	0.0009	0.0009	0.0661	*8.41* × *10^−6^*	*2.443* × *10^−5^*
**Low P Si 0%**	**End**		/	/	0.0026	/	0.0005	/	0.0038	/	6.122 × 10^−6^	/	2.474 × 10^−6^	/	*1.099* × *10^−5^*
**Low P Si 1%**	**Beg.**			/	4.577 × 10^−6^	0.0003	0.0056	0.7684	3.62 × 10^−5^	0.0014	0.0004	0.0067	0.0124	*0.0001*	*0.0004*
**Low P Si 1%**	**End**				/	/	0.0115	/	0.6255	/	0.0004	/	8.033 × 10^−5^	/	*2.649* × *10^−5^*
**Low P Si 10%**	**Beg.**					/	0.0002	0.0004	1.249 × 10^−6^	0.3265	1.739 × 10^−6^	0.9748	2.068 × 10^−5^	*0.0003*	*0.0044*
**Low P Si 10%**	**End**						/	/	0.0401	/	0.6997	/	0.1221	/	*0.0133*
**High P Si 0%**	**Beg.**							/	2.822 × 10^−5^	0.0019	0.0003	0.0089	0.0078	*0.0001*	*0.0004*
**High P Si 0%**	**End**								/	/	0.0017	/	0.0005	/	*0.0018*
**High P Si 1%**	**Beg.**									/	4.484 × 10^−6^	0.595	5.616 × 10^−5^	*0.0003*	*0.0032*
**High P Si 1%**	**End**										/	/	0.0695	/	*5.02* × *10^−5^*
**High P Si 10%**	**Beg.**											/	0.0004	*0.0091*	*0.0769*
**High P Si 10%**	**End**												/	/	*0.0002*
**Peat Control**	**Beg.**													/	*3.663* × *10^−9^*
**Peat Control**	**End**														/

The typesetting of the *p*-values indicated the test in R used for calculation: normal typesetting = *t* test; italic typesetting = welch.test; underlined typesetting = wilcox.test.
